# ﻿A new species of the genus *Podocerus* from the Seto Inland Sea, Japan (Crustacea, Amphipoda, Podoceridae)

**DOI:** 10.3897/zookeys.1128.91155

**Published:** 2022-11-08

**Authors:** Takanobu Arai, Yujiro Ohno, Ko Tomikawa

**Affiliations:** 1 Saijyo Agricultural High School, 3-16-1 Kagamiyama, Higashihiroshima 739-0046, Japan Saijyo Agricultural High School Higashihiroshima Japan; 2 Graduate School of Humanities and Social Sciences, Hiroshima University, 1-1-1 Kagamiyama, Higashihiroshima 739-8524, Japan Hiroshima University Higashihiroshima Japan

**Keywords:** COI, intertidal, podocerid, *Podocerussetouchiensis*, systematics

## Abstract

A new podocerid amphipod, *Podocerussetouchiensis***sp. nov.**, is described from the Etajima Island, the Seto Inland Sea, Japan. This new species differs from its congeners by the dorsal carination of pereonites and pleonites, and form of the antenna 1, gnathopods 1 and 2, uropods 1 and 2, and telson. Nucleotide sequence data of the mitochondrial cytochrome *c* subunit I (COI) from a paratype of *Podocerussetouchiensis***sp. nov.** is provided for future molecular systematic studies.

## ﻿Introduction

*Podocerus* Leach, 1814, is an amphipod crustacean genus belonging to the family Podoceridae Leach, 1814 and is cosmopolitan in world seas (Barnard and Karaman 1991). So far, 63 species of *Podocerus* have been described worldwide (Horton et al. 2022). In Japan, Nagata (1965b) recorded *P.inconspicuus* (Stebbing, 1888) from the sandy mud bottom of the Seto Inland Sea. Hughes (2012) noted morphological differences between Nagata’s (1965b) description of *P.inconspicuus* recorded from Japan and the original description of this species, and considered them to be distinct species. Yamato (1992) described *P.umigame* Yamato, 1992 from green algae growing on the shell of the loggerhead sea turtle *Carettacaretta*. However, this podocerid species is now considered a synonym of *P.chelonophilus* (Chevreux & Guerne, 1888) (Baldinger 2001; Kilgallen 2009; Hughes 2016). Recently, Tomikawa et al. (2019) described *P.jinbe* Tomikawa, Yanagisawa & Vader, 2019, a unique species that lives in the mouth of a whale shark (Tomikawa et al. 2019).

The Seto Inland Sea is the largest inland sea in Japan, surrounded by the three of four largest islands in Japan (excluding Okinawa), Honshu, Shikoku, and Kyushu, with more than 700 islands and a rich marine ecosystem. More than 90 species of amphipods have been reported from the Seto Inland Sea (Nagata 1965a; Hirayama 1987; Tomikawa et al. 2016). During our field survey of shallow-water amphipods in the Seto Inland Sea, an undescribed species of *Podocerus* was collected from coasts of Hiroshima and Okayama Prefectures. In this study, this undescribed species is described and illustrated. In addition, DNA sequence data will be provided for future taxonomic studies based on molecular data.

## ﻿Materials and methods

### ﻿Sampling and morphological observation

**Figure 1.**
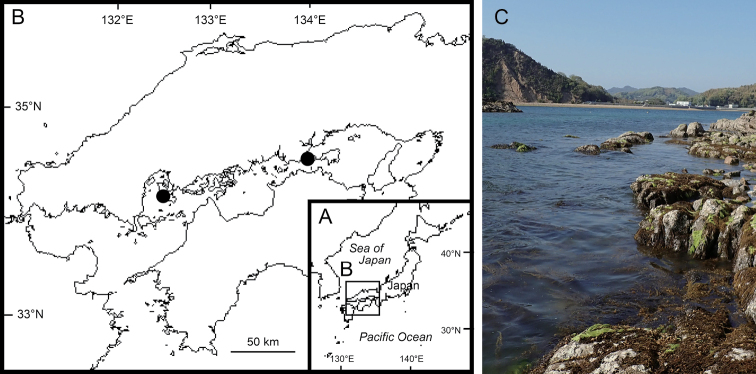
Map showing sampling locality and habitat of *Podocerussetouchiensis* sp. nov. **A** Japan **B** Seto Inland Sea **C** the type locality, Tsuruduki, Etajima, Hiroshima Prefecture, Japan. Circles indicate sampling localities.

Specimens were collected using a hand net and fixed in 99% ethanol on-site (Fig. 1). Some specimens were frozen, then fixed and preserved with polyvinyl alcohol.

All appendages were dissected using insect pins in 80% ethanol and mounted in gum-chloral medium on glass slides using a stereomicroscope (Olympus SZX7). Slides were examined using a light microscope (Nikon Eclipse Ni), with appendages illustrated using a camera lucida. Bodies were dehydrated through a graded ethanol series, and dried using hexamethyldisilazane (HMDS) (Nation 1983). They were then sputter coated with gold and observed using scanning electron microscopy (SEM, JSM-6510LV). Body length was measured from the rostrum tip to the telson base, along the dorsal curvature to the nearest 0.1 mm. The specimens have been deposited in the National Museum of Nature and Science, Tsukuba (**NSMT**).

### ﻿PCR and DNA sequencing

Genomic DNA extraction from body or appendage muscle followed Tomikawa et al. (2014). The cytochrome c oxidase subunit I (COI) gene [LCO1490 and HCO2198 (Folmer et al. 1994)] primer set was used for PCR and cycle sequencing reactions. PCR reactions and DNA sequencing were performed following Tomikawa et al. (2017). Sequences obtained from both strands of the gene segments were edited using MEGA11 (Tamura et al. 2021). DNA sequences have been deposited with the International Nucleotide Sequence Database Collaboration (INSDC) through the DNA Data Bank of Japan (DDBJ).

## ﻿Systematics

### ﻿Suborder Senticaudata Lowry & Myers, 2013

**Infraorder Corophiida Leach, 1814**

**Superfamily Caprelloidea Leach, 1814**

**Family Podoceridae Leach, 1814**

#### Genus *Podocerus* Leach, 1814

##### ﻿ Podocerus setouchiensis sp. nov.

Taxon classificationAnimaliaAmphipodaPodoceridae

00A1D4C7-0992-5BD0-BA52-DBFFE5433F08

https://zoobank.org/0A9432E7-8420-431C-8FA8-243EC74DFDF3

**Figure 2.**
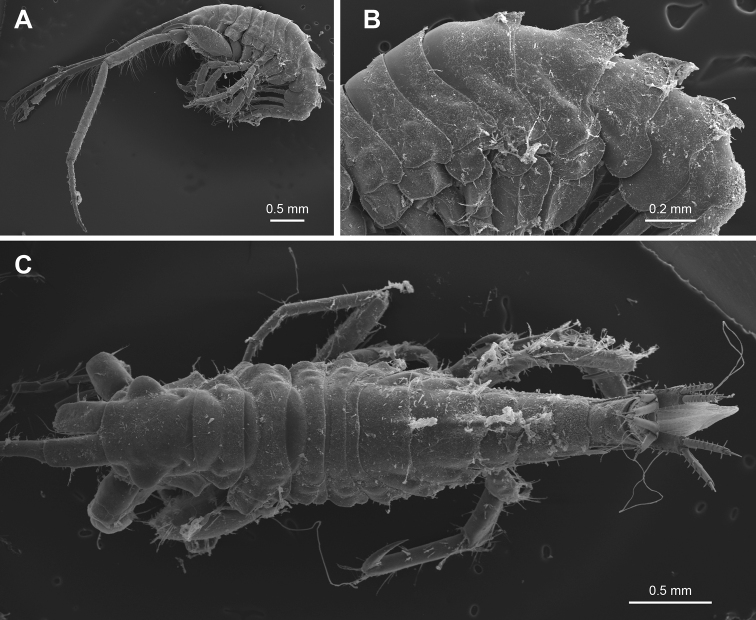
SEM photographs of *Podocerussetouchiensis* sp. nov. **A** habitus, lateral view (NSMT-Cr 30869, male 4.7 mm) **B** dorsal part of pereonites and pleonites, lateral view (NSMT-Cr 30869, male 4.7 mm) **C** habitus, dorsal view (NSMT-Cr 30870, male 4.2 mm).

**Figure 3.**
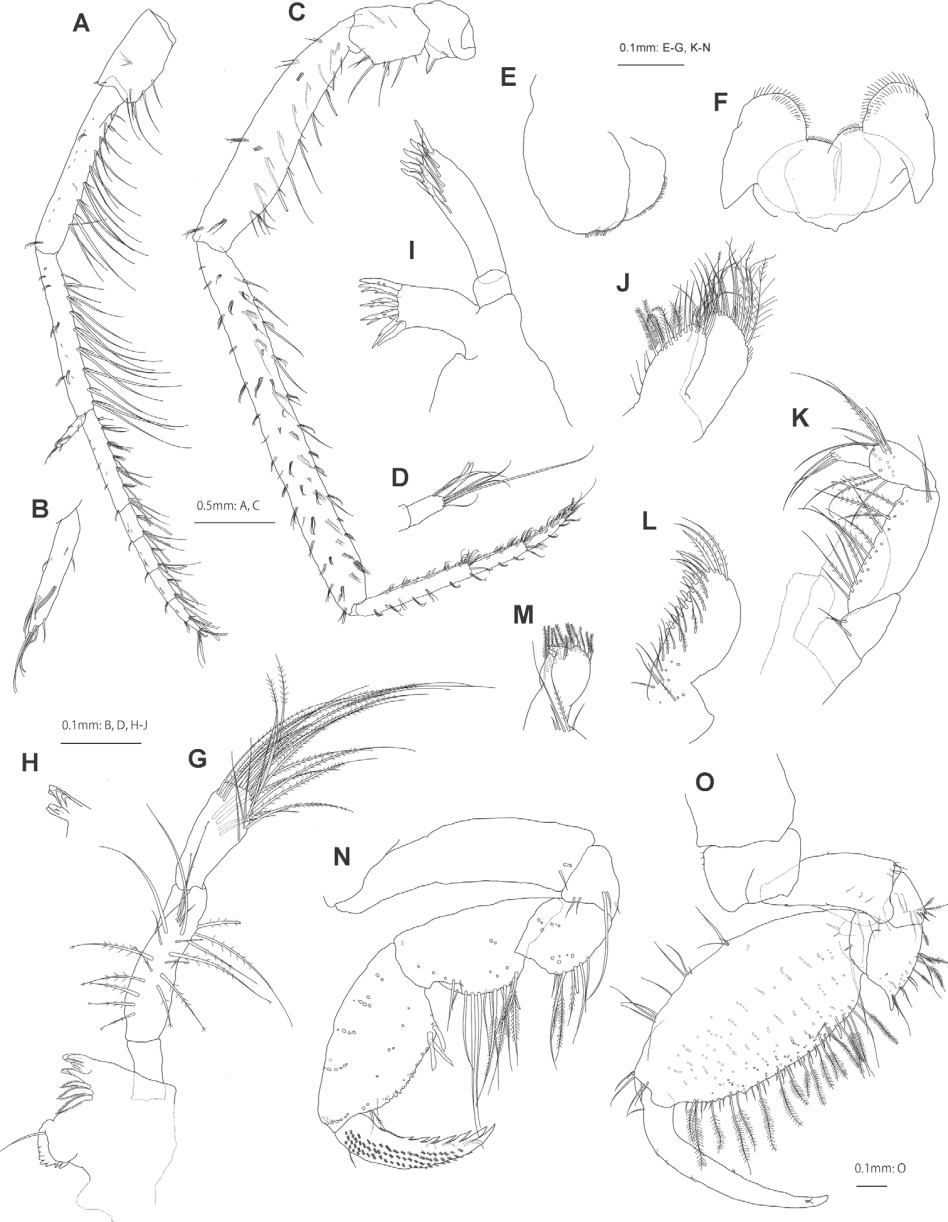
*Podocerussetouchiensis* sp. nov., holotype male, NSMT-Cr 30866 **A** antenna 1, medial view **B** accessory flagellum of antenna 1, medial view **C** antenna 2, medial view **D** distal part of antenna 2, medial view **E** upper lip, anterior view **F** lower lip, posterior view **G** right mandible, medial view **H** incisor and lacinia mobilis of left mandible, lateral view **I** maxilla 1, medial view **J** maxilla 2, medial view **K** palp of maxilliped, medial view **L** outer plate of maxilliped, medial view **M** inner plate of maxilliped, medial view **N** gnathopod 1, lateral view (coxa omitted) **O** gnathopod 2, lateral view.

**Figure 4.**
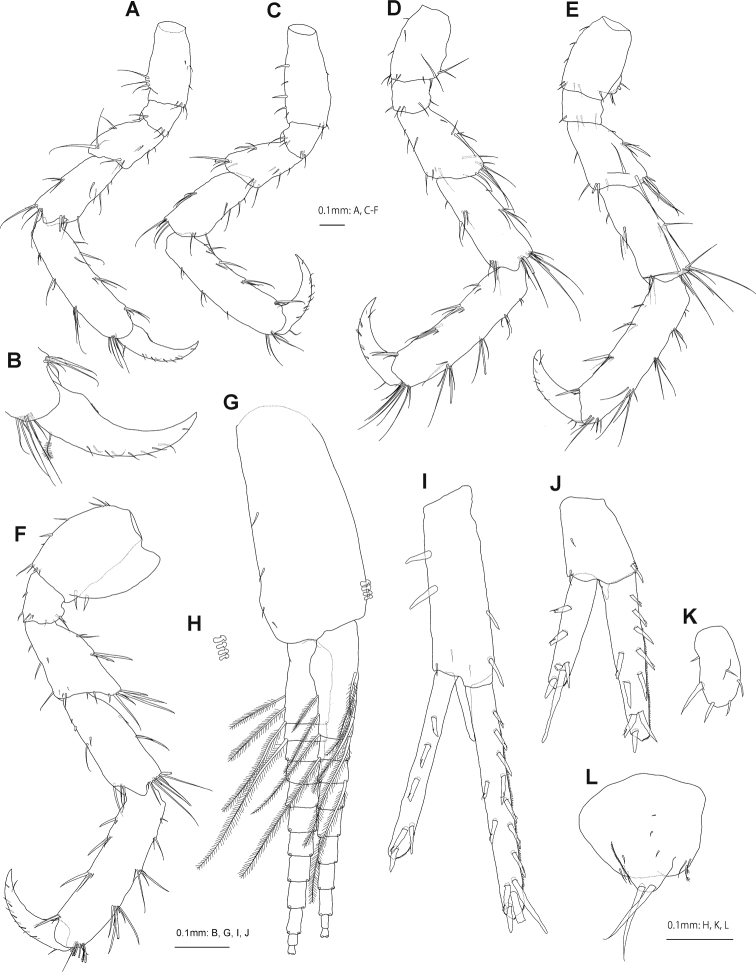
*Podocerussetouchiensis* sp. nov., holotype male, NSMT-Cr 30866 **A** pereopod 3, lateral view (coxa omitted) **B** dactylus of pereopod 3, lateral view **C–F** pereopods 4–7, lateral views (coxae omitted) **G** pleopod 1, posterior view **H** retinacula of pleopod 1, posterior view **I–K** uropods 1–3, dorsal views **L** telson, dorsal view.

**Figure 5.**
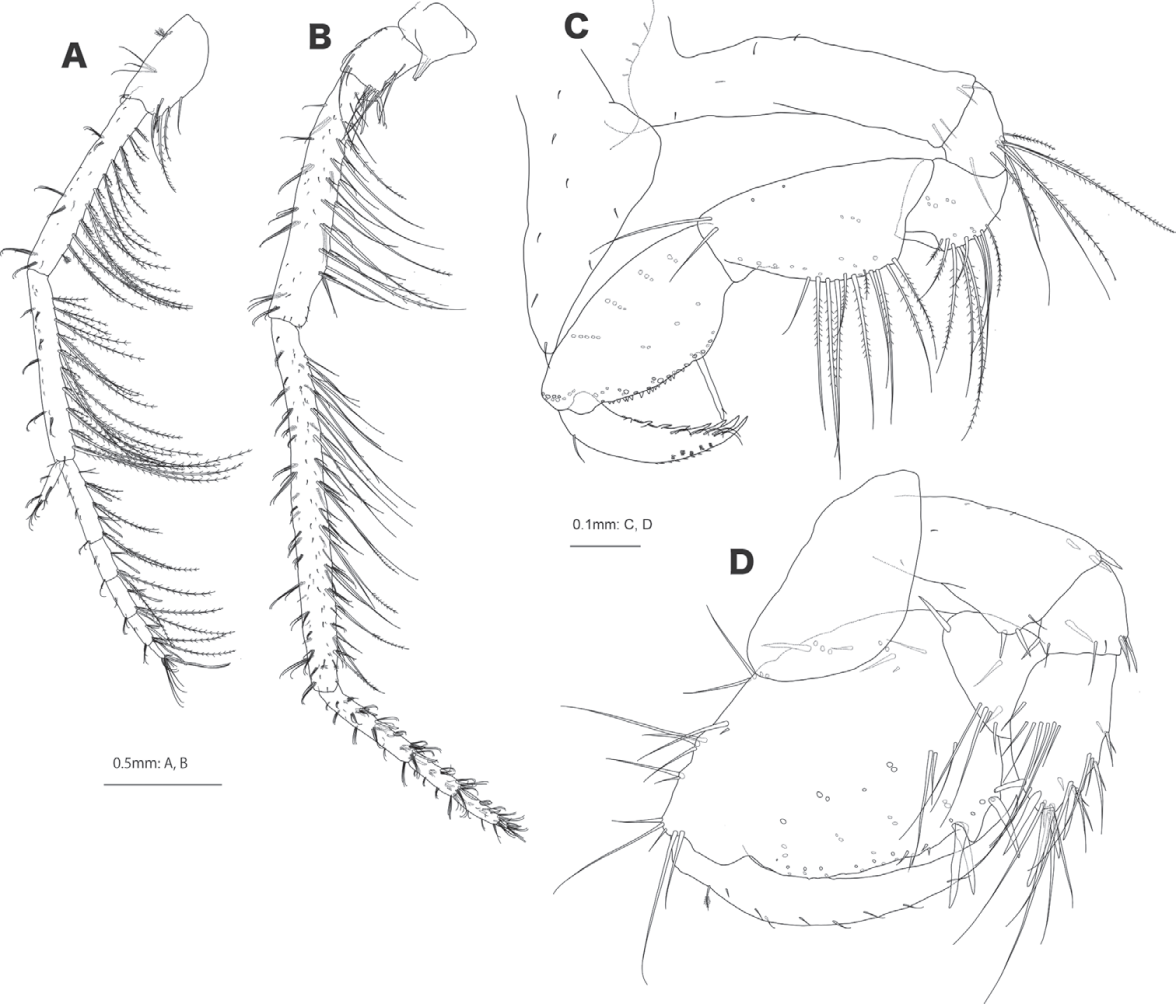
*Podocerussetouchiensis* sp. nov., paratype female, NSMT-Cr 30867 **A** antenna 1, medial view **B** antenna 2, medial view **C** gnathopod 1, lateral view **D** gnathopod 2, lateral view.

Figs 2 , 3 , 4 , 5 New Japanese name: Setouchi-doronomi

###### Material examined.

***Holotype***: NSMT-Cr 30866, male (5.3 mm), intertidal zone of Tsuruduki, Etajima, Hiroshima Prefecture, Japan (34.1462°N, 132.4399°E), collected by K. Tomikawa on 18 April 2019. ***Paratypes***: NSMT-Cr 30867 (female 4.6 mm), NSMT-Cr 30868 (male 6.0 mm, G1527), NSMT-Cr 30869 (male 4.7 mm), NSMT-Cr 30870 (male 4.2 mm), data same as for holotype; NSMT-Cr 30871, 2 males (5.9 mm, 5.1 mm), intertidal zone of Gokan, Tamano, Okayama Prefecture, Japan (34.5263°N, 133.9893°E), collected by H. Ogawa on 9 April 2019.

###### Diagnosis.

Body weakly rugose; pereonites 6–7 and pleonites 1–2 with dorsal carina. Head dorsally smooth. Antenna 1 accessory flagellum 1-articulate. Antenna 2 flagellar article 1 elongate. Uropod 3 rami with setae. Telson shorter than wide, with 2 long robust setae apically, lower margin with short lateral setae.

###### Description

**(male, holotype, NSMT-Cr 30866).** Body (Fig. 2A–C) weakly rugose; pereonites 6–7 and pleonites 1–2 with dorsal carina. Head (Fig. 2A, C) dorsally smooth; rostrum absent; lateral cephalic lobe squarish. Gnathopod 2 palm of propodus slightly convex with long plumose setae. Uropods 1 and 2 with distoventral projection.

Antenna 1 (Fig. 3A, B) length 0.8× body length; length ratio of peduncular articles 1–3 1.0: 2.1: 2.0; peduncular article 1 subquadrate, with long setae on posterior margin; peduncular articles 2 and 3 with 9 and 8 clusters of long setae on posterior margins, respectively; primary flagellum 6-articulate, 0.6× peduncular articles 1–3 combined, article 1 long, 3.2× article 2; accessory flagellum slender, 1-articulate. Antenna 2 (Fig. 3C, D) 1.5× antenna 1; peduncular article 4 with long setae on posterior margin; peduncular article 5, 1.4× article 4, with short setae on anterior and posterior margins; flagellum 4-articulate, 0.2× peduncular articles 1–5 combined, article 1 slightly longer than articles 2–4 combined.

Upper lip (Fig. 3E) oval, ventral margin weakly concave, with minute setae. Lower lip (Fig. 3F) outer lobe broad, setulose; inner lobes distinct. Left and right mandibles (Fig. 3G, H) with 5-dentate incisor; molar process small, non-triturative, with a short plumose seta apically; accessory setal row with 3 setae; palp 3-articulate, length ratio of articles 1–3 1.0: 2.7: 1.9, article 1 bare, article 2 with 20 setae on ventral margin and submargin, ventral margin of article 3 lined with plumose setae, inner surface of article 3 with cluster of setae. Maxilla 1 (Fig. 3I) inner plate indistinct; outer plate rectangular with 9 serrate robust setae; palp article 2 bearing 5 robust and 6 slender setae distally. Maxilla 2 (Fig. 3J) with broad outer plate longer than inner plate, inner and outer plates bearing long plumose setae on apical margin. Maxilliped (Fig. 3K–M) inner plate subrectangular, 3 small robust setae on apical margin and 1 on subapical margin; outer plate slightly exceeding half of palp article 2, medial margin with robust setae and long plumose setae; palp 4-articulate.

Gnathopod 1 (Fig. 3N) coxa slender, subtriangular, longer than broad; basis length 3.7× width, lacking setae on anterior and posterior margins; carpus 2.0× broad, ventral margin weakly lobate with long setae; propodus subtriangular, length 1.9× wide, anterior margin with 4 clusters of slender setae, posterior margin convex with 3 robust setae at palmar corner; posterior margin of dactylus 6-dentate with short setae. Gnathopod 2 (Fig. 3O) coxa quadrate; basis 1.9× broad, weakly concave anteriorly, anterodistal corner lobate; posterodistal corner of merus produced with simple and plumose setae; carpus indistinct, fused with propodus, with setae; propodus subovate, 2.1× wide, anterior margin with 3 clusters of setae and single seta, medial surface with numerous plumose setae, palm slightly convex with long plumose setae, distal shelf well-developed, robust seta at palm defining corner; dactylus smooth, not reaching end of palm, with short setae.

Pereopods 3 and 4 (Fig. 4A–C) basis lacking anterodistal lobe; ischium subrectangular; merus slightly shorter than carpus, anterodistal corner weakly produced; propodus longer than carpus, lacking robust setae marginally. Pereopod 5 (Fig. 4D) basis without posterodistal lobe; posterodistal corner of merus weakly produced; length ratio of merus-dactylus 1.0: 1.2: 1.8: 1.0. Pereopod 6 (Fig. 4E) basis subrectangular, length 1.4× wide, posterodistal corner weakly lobate; posterodistal corner of merus weakly produced; length ratio of merus-dactylus 1.0: 1.2: 2.0: 0.9. Pereopod 7 (Fig. 4F) basis length 1.2× width, posterior margin expanded; merus produced posterodistally; length ratio of merus-dactylus 1.0: 1.2: 1.7: 1.2.

Pleopods 1–3 (Fig. 4G) peduncle with short setae, inner distal corner with 4 retinacula (Fig. 4H).

Uropod 1 (Fig. 4I) biramous; peduncle 3.4× broad, medial and lateral margins each with 4 robust setae, distoventral projection length 0.3× peduncle; inner ramus 1.2× peduncle, with 7 medial and 2 lateral robust setae; outer ramus 0.8× length of inner ramus, with 3 robust setae on lateral margin. Uropod 2 (Fig. 4I) biramous; peduncle 1.3× broad, with short distoventral projection; inner ramus 1.9× peduncle, medial and lateral margins with 5 and 2 robust setae, respectively; outer ramus 0.7× length of inner ramus, bearing 2 lateral robust setae. Uropod 3 (Fig. 4K) uniramous, plate-like; with 4 apical, 1 medial and 1 lateral robust setae.

Telson (Fig. 4L) length 0.9× width, dorsal lobe with 2 long robust setae apically, lower margin with short lateral setae.

**Female (paratype, NSMT-Cr 30867).** Antenna 1 (Fig. 5A) length ratio of peduncular articles 1–3 1.0: 2.1: 1.9; primary flagellum 5-articulate, 0.4× peduncular articles 1–3 combined, article 1 long, 2.0× article 2. Antenna 2 (Fig. 5B) peduncular article 5, 1.4× article 4; flagellum 3-articulate, 0.3× peduncular articles 1–5 combined.

Gnathopod 1 (Fig. 5A) basis almost straight; carpus 2.3× broad; palmar margin of propodus bearing robust seta; posterior margin of dactylus 4-dentate. Gnathopod 2 (Fig. 5B) basis with anterodistal robust setae; merus produced anterodistally with robust setae; carpus free, distinct from propodus; propodus ovate, length 1.4× width, palm convex with 3 robust setae near palmar corner.

###### Etymology.

The specific name is derived from the Seto Inland Sea, where this new species is distributed.

###### DNA Sequence.

A sequence of COI (GenBank accession number LC719250; 658 bp) was determined from the paratype female (NSMT-Cr 30868).

###### Distribution.

Known from Hiroshima and Okayama Prefectures.

###### Remarks.

*Podocerussetouchiensis* sp. nov. is similar to *P.andamanensis* (Giles, 1890), *P.casuarinensis* Kilgallen, 2009, *P.crenulatus* Myers, 1985, *P.fulanus* J.L. Barnard, 1962, *P.lazowasemi* Baldinger & Gable, 1994, *P.orontes* Hughes, 2013, and *P.walkeri* Rabindranath, 1972 in having dorsal carinae on pereonites 6 and 7, and pleonites 1 and 2. However, this new species differs from these species by the features shown in the following key. *Podocerussetouchiensis* sp. nov. is also similar to *P.ulreungensis* Kim & Kim, 1991 from Ulleung Island in the Sea of Japan in having antenna 2 with elongate flagellar article 1, gnathopod 1 with weakly lobate carpus, male gnathopod 2 merus pointed distally, male gnathopod 2 propodus with convex palmar margin bearing long marginal setae and robust seta at palm defining corner, uropod 1 peduncle with distoventral projection, and telson with 2 apical setae. However, this new species is distinguished from the latter by the following features (features of *P.ulreungensis* in parentheses): pereonite 6 with dorsal carina (absent); antenna 1 accessory flagellum 1-articulate (2-articulate); antenna 1 flagellar article 1 length 3.2 times as long as article 2 (2.3 times); uropod 2 peduncle with a short distoventral projection (lacking projection); and telson shorter than wide (longer).

### ﻿Key to species of *Podocerus* with dorsal carina on pereonites 6 and 7 (lacking dorsal carina on pereonites 1–5).

**Table:** 

1	Uropods 1 and 2 with peduncular distoventral projection	**2**
–	Uropods 1 and 2 without peduncular distoventral projection	**5**
2	Telson with 2 apical setae	***P.setouchiensis* sp. nov.**
–	Telson with 4 or 5 apical setae	**3**
3	Male gnathopod 2 palmar margin of propodus concave, with long plumose setae, lacking robust setae; uropod 3 without setae on rami	***P.orontes* Hughes, 2013**
–	Male gnathopod 2 palmar margin of propodus convex, with short slender and robust setae; uropod 3 with setae on rami	**4**
4	Coxa of gnathopod 1 triangular; gnathopod 2 palmar margin of propodus with robust setae on proximal corner	***P.fulanus* J.L. Barnard, 1962**
–	Coxa of gnathopod 1 subquadrate; gnathopod 2 palmar margin of propodus lined with robust setae	***P.lazowasemi* Baldinger & Gable, 1994**
5	Antenna 1 accessory flagellum 2-articulate	***P.crenulatus* Myers, 1985**
–	Antenna 1 accessory flagellum 1-articulate	**6**
6	Uropod 1 inner ramus marginally bare	***P.casuarinensis* Kilgallen, 2009**
–	Uropod 1 inner ramus with marginal robust setae	**7**
7	Male gnathopod 1 palmar margin of propodus almost straight	***P.andamanensis* (Giles, 1890)**
–	Male gnathopod 1 palmar margin of propodus convex	***P.walkeri* Rabindranath, 1972**

## Supplementary Material

XML Treatment for Podocerus setouchiensis
